# Enhancing rigor and reproducibility: Implementing STAR Methods and the ISSCR Standards Checklist at Stem Cell Reports

**DOI:** 10.1016/j.stemcr.2026.102817

**Published:** 2026-02-12

**Authors:** Janet Rossant

**Affiliations:** 1 Editor-in-Chief, Stem Cell Reports

## Main text

As the official journal of the International Society for Stem Cell Research (ISSCR), Stem Cell Reports has always taken very seriously its responsibility to publish stem cell-related research of the highest standards of rigor and reproducibility. Across all areas of scientific research, there is increasing concern that reporting standards are not always transparent and accessible, thus making it difficult for investigators to readily reproduce the work of other laboratories.

Reporting standard guidelines and checklists plays an important part in promoting rigor and reproducibility. With this in mind, Stem Cell Reports is implementing two new requirements for publication in the journal: mandated completion of the ISSCR Standards Checklist prior to review and submission of the paper’s Methods section in the STAR Methods format prior to publication.

### ISSCR Standards for the Use of Human Stem Cells in Research Checklist

In 2023, the ISSCR Standards Task Force published its guidelines document, Standards for the Use of Human Stem Cells in Research, https://www.isscr.org/basic-research-standards/#online (Ludwig et al., 2023). It provided a set of recommendations to help establish the minimum characterization and reporting criteria expected for laboratories working with human stem cells, whether pluripotent or tissue specific. In October 2023, Stem Cell Reports introduced the ISSCR Standards Checklist based on these standards. While it was required to be completed when submitting a manuscript describing primary research involving human stem cells, to date, the checklist was only scanned editorially and was not sent to the reviewers as part of the editorial process. In this issue of Stem Cell Reports, Fischer et al. (2026) provide a survey of a small, anonymized sample of the submitted checklists and find that in many cases, the checklist is incomplete and the associated information is missing in the paper. Fischer et al. provide advice to researchers on how to generate and record this essential information. In addition, ISSCR and Stem Cell Technologies have partnered to deliver a free course with practical guidance on implementing the ISSCR standards: https://www.isscr.org/applying-research-standards-course. Given all this guidance, and the importance of these standards for rigor and reproducibility, as of April 1, 2026, Stem Cell Reports will include the authors’ completed ISSCR Standards Checklist with the material provided to reviewers. Reviewers will be asked to comment on whether the checklist and associated information in the manuscript provide sufficient details to assure rigor and reproducibility of the data reported in the paper. Authors may be requested to provide additional information or data as necessary. We hope that over time this effort will lead to a continued improvement in reporting standards for stem cell research.

### STAR Methods

While the Standards Checklist addresses issues specific to stem cells, there is also a strong movement toward standardization and transparency of reporting across all aspects of experimental science. To this end, Cell Press, the publisher of Stem Cell Reports, has implemented STAR Methods, a standardized format for reporting on the methods, reagents, and techniques used in any published study. STAR stands for “structured, transparent, accessible reporting.” STAR Methods has no length limitations for the methods write-up ensuring that full details of methodologies can be provided. Most importantly there is a single list, the key resources table, of all items necessary to reproduce the research results contained in the paper and their sources: https://www.cell.com/information-for-authors/star-authors-guide.

We believe that STAR Methods, which many of you are probably already familiar with, can be implemented relatively easily. The key resources table, in particular, is an incredibly useful resource for everyone. Thus, as of April 1, 2026, all papers published in Stem Cell Reports will be required to provide their Materials and Methods section in the extended STAR Methods format. Your manuscript does not have to adhere to the STAR Methods format at the time of submission, but adhering to this format early in the submission process is highly recommended. The editorial team will require reformatting to the STAR Methods format prior to any final acceptance.

There will likely be some overlap between information requested in the ISSCR Standards checklist and the STAR Methods. The final STAR Methods section is expected to encompass all this information and will provide a robust resource to accompany your papers.

I hope you will support us in promoting ongoing efforts to enhance rigor and reproducibility in stem cell research.
